# Hirsutine induces mPTP-dependent apoptosis through ROCK1/PTEN/PI3K/GSK3β pathway in human lung cancer cells

**DOI:** 10.1038/s41419-018-0641-7

**Published:** 2018-05-22

**Authors:** Rong Zhang, Guobing Li, Qian Zhang, Qin Tang, Jingbin Huang, Changpeng Hu, Yali Liu, Qing Wang, Wuyi Liu, Ning Gao, Shiwen Zhou

**Affiliations:** 1 Department of Pharmacy, The Second Affiliated Hospital, Army Medical University, 400037 Chongqing, China; 2 College of Pharmacy, Army Medical University, 400038 Chongqing, China

## Abstract

Hirsutine extracted from *Uncaria rhynchophylla* has been shown to exhibit anti-cancer activity. However, the molecular mechanism by which hirsutine exhibits anti-lung cancer activity remains unclear. In the present study, we showed that hirsutine induces apoptosis in human lung cancer cells via loss of mitochondrial membrane potential (∆*ψ*m), adenosine triphosphate (ATP) depletion, ROS production, as well as cytochrome c release. Dephosphorylation of GSK3β is involved in hirsutine-mediated mitochondrial permeability transition pore (mPTP) opening through ANT1/CypD interaction. Mechanistic study revealed that interruption of ROCK1/PTEN/PI3K/Akt signaling pathway plays a critical role in hirsutine-mediated GSK3β dephosphorylation and mitochondrial apoptosis. Our in vivo study also showed that hirsutine effectively inhibits tumor growth in a A549 xenograft mouse model through ROCK1/PTEN/PI3K/Akt signaling-mediated GSK3β dephosphorylation and apoptosis. Collectively, these findings suggest a hierarchical model in which induction of apoptosis by hirsutine stems primarily from activation of ROCK1 and PTEN, inactivation of PI3K/Akt, leading in turn to GSK3β dephosphorylation and mPTP opening, and culminating in caspase-3 activation and apoptosis. These findings could provide a novel mechanistic basis for the application of hirsutine in the treatment of human lung cancer.

## Introduction

**Fig. 1:**
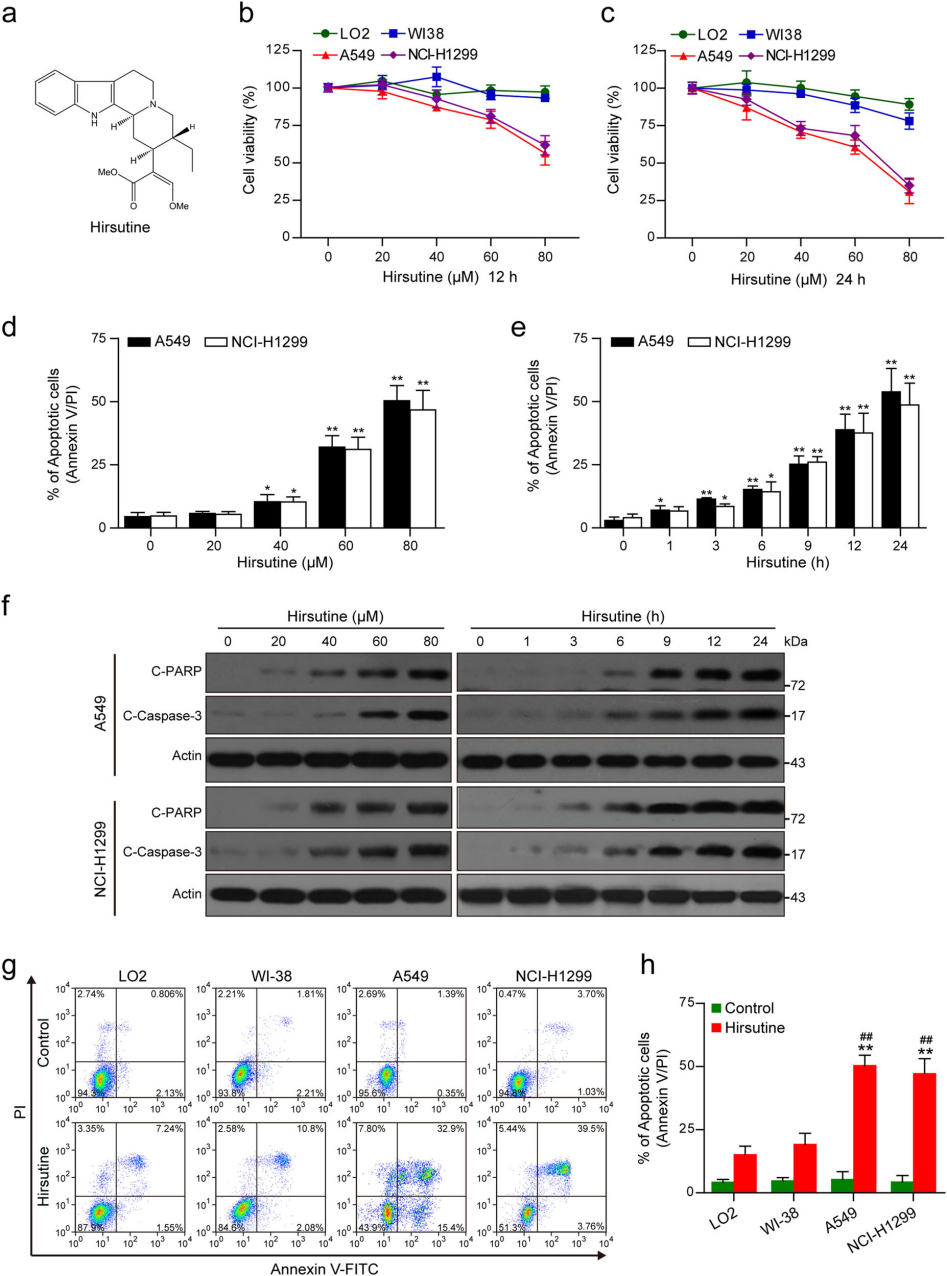
Hirsutine selectively inhibits cell proliferation and induces apoptosis in human lung cancer cells. **a** The chemical structure of hirsutine. **b**, **c** Human normal hepatocyte LO2 cells, human lung epithelial fibroblast WI38 cells, and human lung carcinoma A549 and NCI-H1299 cells were treated with various concentrations of hirsutine for 12 or 24 h; cell viabilities were determined by Cell Counting Kit-8 (CCK-8). Data are expressed as the mean ± SD (*n* = 3). **d**, **e** A549 and NCI-H1299 cells were treated with various concentrations of hirsutine for 24 h or 80 μM hirsutine for different time intervals. The percentage of apoptotic cells was determined by flow cytometry using Annexin V-FITC/PI staining. Data are expressed as the mean ± SD (*n* = 3). *P < 0.05, **P < 0.01 vs. the control group. **f** Whole-cell lysates were prepared and subjected to western blot analysis using the indicated antibodies against cleaved-PARP (C-PARP), cleaved-caspase 3 (C-Caspase 3). **g**, **h** LO2, WI38, A549, and NCI-H1299 cells were treated with 80 μM hirsutine for 24 h, followed by staining with Annexin V-FITC/PI and analyzed by flow cytometry. Data are expressed as the mean ± SD (*n* = 3), **P < 0.01 vs. LO2 cells treated with hirsutine, ^##^*P* < 0.01 vs. WI-38 cells treated with hirsutine

Phytochemicals are promising sources for the development of novel cancer therapeutics. Due to their potential efficiency and low toxicity profiles^1,2^, phytochemicals have generally been successful for developing the novel agents to treat many diseases^3,4^. *Uncaria rhynchophylla* is a traditional oriental herb that has been used as spasmolytics, analgesics, and sedatives, also used to treat various cerebrovascular diseases, epilepsy, stroke, preeclampsia, and nervous disorders^5^. Hirsutine, a major indole alkaloid (Fig. 1a), derived from *U. rhynchophylla*, has attracted a great deal of interest because of its wide range of biological activities such as cardioprotective, anti-hypertensive, and anti-arrhythmic activities^6,7^. It has recently been shown that hirsutine exhibits anticancer activities. Hirsutine selectively induces apoptotic cell death in multiple human breast cancer cell lines, and the mechanistic study showed that suppression of HER2, NF-κB, and Akt pathways and activation of the p38 MAPK pathway could be involved in hirsutine-induced DNA damage and apoptosis^8^. It has also been reported that hirsutine potentially inhibited metastasis in 4T1 breast cancer cells both in vitro and in vivo through interruption of NF-κB signaling pathway^9^. However, little is known about other signaling pathways involved in the regulation of hirsutine-induced apoptosis. Also, the molecular mechanism by which hirsutine regulates the mitochondrial pathway of apoptosis in human lung cancer cells has not yet been explored.

Mitochondria play a critical role in mediating intrinsic pathway of apoptosis in mammalian cells. A number of studies have uncovered that the dysfunction of mitochondria upon apoptotic stimuli is controlled by mitochondrial outer membrane permeabilization (MOMP), which is regulated by two pro-apoptotic Bcl-2 family proteins, Bax and Bak. The pro-apoptotic Bcl-2 proteins (Bad, Bid, Bim, Noxa, Puma) are considered the sentinels for the stress signals, increase MOMP by directly or indirectly activating Bax/Bak, but these direct activators are sequestered by the anti-apoptotic Bcl-2 proteins (Bcl-2, Bcl-xL, Mcl-1)^10–12^. Mitochondrial permeability transition pore (mPTP) is another key participant in mitochondrial apoptosis^13^. mPTP opening has catastrophic consequences on the fate of cells, leading to release of Ca^2+^ from the mitochondrial matrix, adenosine triphosphate (ATP) depletion, reactive oxygen species (ROS) production, and release of pro-apoptotic factors (i.e., cytochrome c) that trigger caspases activation and apoptosis^14^. mPTP is primarily composed of voltage-dependent anion channel (VDAC), adenine nucleotide translocator 1 (ANT1), and cyclophilin-D (CypD)^15^. Under stress, CypD binding with ANT1 in the inner mitochondrial membrane (IMM), leading to mPTP opening^16^. It has been shown that glycogen synthase kinase-3β (GSK3β) plays a role in regulating mPTP opening; physical interaction of phospho-GSK3β with ANT1 suppresses interaction of ANT1 with CypD, resulting in an increase in the threshold for mPTP opening^17,18^.

Rho-associated coiled-coil kinase (ROCK) belongs to a family of serine/threonine kinases that are activated via interaction with Rho GTPases. ROCK is involved in a wide range of fundamental cellular functions, such as adhesion, migration, proliferation, and apoptosis^19^. Phosphatase and tensin homolog (PTEN) is an important ROCK substrate, the phosphorylation of PTEN by ROCK stimulates its phosphatase activity^20,21^. PTEN dephosphorylates both proteins and phosphoinositides and is a negative regulator of the phosphatidylinositol (PI)3-kinase/Akt pathway, which has important roles in a diverse range of biological processes, including cell survival and cell death^22^. However, the precise mechanism by which ROCK1 regulates the phosphorylation states of PTEN/PI3K/Akt during hirsutine-mediated apoptosis in human lung cancer cells is unclear.

In the present study, we investigated how hirsutine induces apoptosis in human lung cancer cells. Specifically, we evaluated the role of the ROCK1/PTEN/PI3K/Akt signaling pathway in regulating mitochondrial apoptosis. We found that hirsutine induces mitochondrial apoptosis in human lung cancer cells and inhibits tumor growth of A549 xenograft mouse model. Mechanistic studies revealed that interruption of the ROCK1/PTEN/PI3K/Akt signaling pathway plays a critical role in hirsutine-mediated dephosphorylation of GSK3β, opening of mPTP, depletion of ATP, and induction of mitochondrial apoptosis ultimately. Our study provides novel insight into apoptotic effects of hirsutine and suggests that hirsutine may be a valuable chemotherapeutic agent for the clinical treatment of human lung cancer.

## Results

### Hirsutine selectively inhibits cell proliferation and induces apoptosis in human lung cancer cells

First, we evaluated the effects of hirsutine on cell proliferation in human non-small cell lung cancer (NSCLC) cells (A549 and NCI-H1299), normal human lung fibroblast WI38 cells, and human normal LO2 hepatocytes. Exposure of A549 and NCI-H1299 cells to hirsutine resulted in a significant decrease in cell viability in a dose- and time-dependent manner. In contrast, hirsutine exerted little inhibitory effects toward normal human lung fibroblast WI38 cells and human normal LO2 hepatocytes after 12 and 24 h of drug exposure (Fig. 1b, c). To determine whether apoptosis induction contributes to inhibition of cell proliferation mediated by hirsutine, flow cytometry analysis was employed. Exposure of A549 and NCI-H1299 cells to hirsutine resulted in a significant increase in apoptosis in a dose- and time-dependent manner (Fig. 1d, e). Consistent with these findings, the same hirsutine concentrations and exposure intervals resulted in cleavage/activation of caspases-3 and degradation of PARP (Fig. 1f). However, hirsutine regimen induced relatively little apoptosis in normal human lung fibroblast WI38 cells and human normal LO2 hepatocytes (Fig. 1g, h). Together, these findings indicate that hirsutine selectively inhibits cell proliferation and induces caspase-dependent apoptosis in human lung cancer cells but not normal lung fibroblast cells and normal hepatocytes.

### Hirsutine induces loss of Δψm, ATP depletion, ROS production, as well as cytochrome c release

**Fig. 2:**
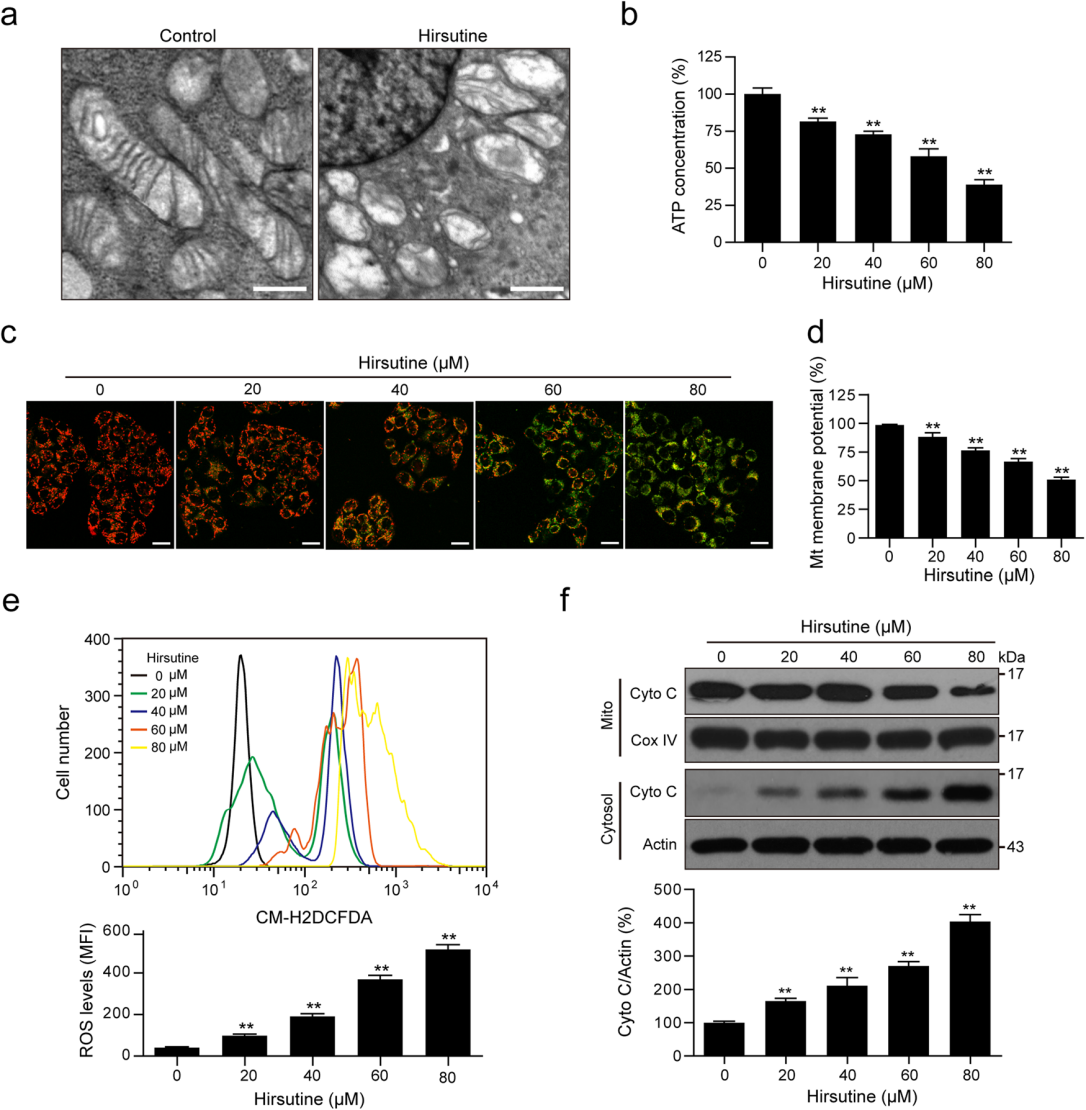
Hirsutine induces mitochondrial injury in A549 cells. **a** A549 cells were treated with 80 μM hirsutine for 24 h, mitochondria were observed by transmission electron microscopy. Scale bars: 1 μm. **b** A549 cells were treated with various concentrations of hirsutine for 24 h. ATP concentrations were determined using an ATP Determination Kit. **c**, **d** Mitochondrial membrane potential as analyzed by confocal microscopy or microplate reader using JC-1 staining. Scale bars: 20 μm. Ratio of red/green fluorescence intensity represents the potential of the mitochondrial membrane. **e** Intracellular ROS level was detected by DCF-DA staining; mean value of ROS fluorescence intensity was measured by flow cytometry. **f** Mitochondrial (Mito) and cytosolic (Cytosol) fractions were prepared and subjected to western blot analysis. The relative intensities of Cyto C in cytosolic fractions were normalized to Actin by densitometric analysis using Quantity One software. Data are expressed as the mean ± SD (*n* = 3). The results were expressed as a percentage of control, which was set at 100%, **P < 0.01 vs. the control group

Increasing evidences indicate that mitochondrial dysfunction has been shown to participate in the induction of apoptosis^23–25^. We next observed the effects of hirsutine on the morphology of mitochondria by using electron microscopic analysis. In control cells, the majority of mitochondria had normal structures with intact membranes and cristae. Cells treated with hirsutine showed typical morphological changes of damage, mitochondrial swelling, rupture of the mitochondrial outer membrane, and distorted mitochondrial cristae (Fig. 2a). Mitochondria play a crucial role in most of the cellular energy ATP production, and mitochondrial dysfunction is always accompanied with ATP depletion^26–28^. We then examined the effects of hirsutine on the content of ATP. Treating cells with hirsutine resulted in significant decreases in levels of ATP in a dose-dependent manner (Fig. 2b).

Loss of mitochondrial membrane potential (∆*ψ*m) is another indicator of mitochondrial dysfunction^29,30^. We employed a mitochondrial ∆*ψ*m-sensitive dye JC-1 to detect the mitochondrial membrane potential. The high ∆*ψ*m of control cells loaded with JC-1 allows for the formation of red-fluorescent J-aggregates. Upon loss of ∆*ψ*m, these J-aggregates dissipate into monomers leading to a shift from red to green fluorescence. The dose-dependent decreases in red fluorescence and increases in green fluorescence were observed in cells treated with hirsutine (Fig. 2c). By using fluorescent microplate reader, we also found that the red/green fluorescence intensity ratio was significantly decreased after hirsutine treatment (Fig. 2d). A dose-dependent increase in ROS production was also observed after hirsutine treatment (Fig. 2e). Western blot analysis showed that treating cells with hirsutine resulted in dose-dependent increases in the release of cytochrome c from the mitochondria into the cytosol (Fig. 2f). Taken together, these findings indicate that hirsutine induces mitochondrial apoptosis via loss of Δ*ψ*m, ATP depletion, ROS production, as well as cytochrome c release.

### The association between CypD and ANT1 is involved in hirsutine-induced mPTP opening and apoptosis

The Bcl-2 family proteins are the best characterized regulators of apoptosis, including the anti-apoptotic members (Bcl-2, Bcl-xL, Mcl-1), BH3-only proteins (Bad, Bid, Bim, Noxa, Puma), and pro-apoptotic members (Bax, Bak)^10^. We next examined the effects of hirsutine on the expression of these Bcl-2 family proteins. Treatment of cells with hirsutine resulted in slight decrease in levels of Bcl-xL. In contrast, exposure of cells to hirsutine did not discernibly modify the expression of other Bcl-2 family proteins, including Bax, Bak, Puma, Bim, Noxa, Mcl-1, and Bcl-2 (Figure S1a). To further confirm the functional role of Bax/Bak in hirsutine-induced apoptosis in human lung cancer cells, two specific shRNA were employed to knockdown the expression of Bax and Bak. Double knockdown of Bax/Bak did not obviously affect hirsutine-induced apoptosis. In contrast, double knockdown of Bax/Bak significantly attenuated etoposide-induced apoptosis (Figure S1b-c). These findings suggest that Bax/Bak may not be critical for hirsutine-induced apoptosis.

**Fig. 3:**
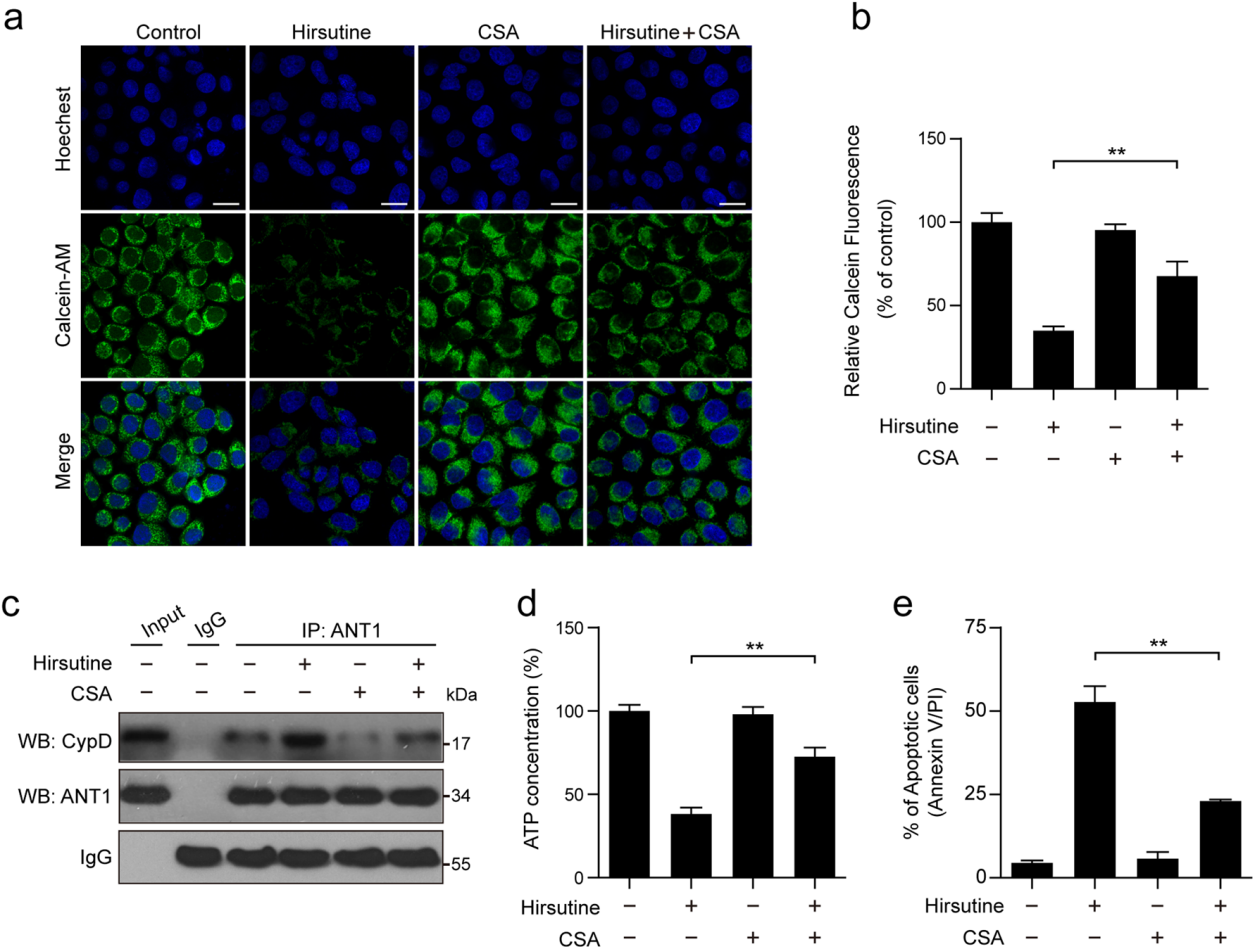
Hirsutine induces apoptosis dependent on mitochondrial permeability transition pore (mPTP) opening. **a**, **b** A549 cells were pretreated with 5 µM cyclosporin A (CSA, a CypD inhibitor) for 2 h, followed by treatment with 80 μM hirsutine for 24 h. Cells were stained with calcein-AM and CoCl_2_ (cytosolic calcein quencher), the calcein fluorescence in the mitochondria was analyzed by confocal laser scanning microscope or microplate reader. Scale bars: 20 μm. Data are expressed as the mean ± SD (*n* = 3). *P < 0.05, **P < 0.01. **c** Cells were treated as indicated in **a**, equal amount of lysates were subjected to immunoprecipitation using anti-ANT1 antibody, the associated CypD was determined using immunoblotting. **d** Cells were treated as indicated in **a**, the ATP concentrations were measured using a firefly luciferase-based ATP Determination Kit. **e** Cells were treated as indicated in **a**, the percentage of apoptotic cells was determined by flow cytometry using Annexin V-FITC/PI staining. Data are expressed as the mean ± SD (*n* = 3), **P < 0.01

Increasing evidence indicates that opening of the mPTP disrupts the permeability barrier of the IMM and leads to mitochondrial swelling, dissipation of mitochondrial membrane potential, depletion of ATP, and increase in ROS generation^31–36^. We next examined the effects of hirsutine on mPTP opening by using calcein AM-cobalt chloride quenching method. A reduction in mitochondrial calcein fluorescence represented the opening of mPTP was observed in cells treated with hirsutine (Fig. 3a). CypD is an essential regulatory component of mPTP^37^, our data showed that cyclosporin A (CSA), a well-known inhibitor of CypD, significantly prevented hirsutine-mediated mPTP opening (Fig. 3a, b). It has been shown that the association between CypD and ANT1 is an initial step of mPTP opening^16,38^. The immunoprecipitation assay showed that treating cells with hirsutine resulted in increase in association between CypD and ANT1, whereas pretreatment with CSA attenuated hirsutine-induced association between CypD and ANT1 (Fig. 3c). Furthermore, CSA pretreatment significantly attenuated hirsutine-mediated ATP depletion and apoptosis (Fig. 3d, e). Taken together, these results suggest that the association between CypD and ANT1 is involved in hirsutine-induced mPTP opening and mitochondrial apoptosis in A549 cells.

### Dephosphorylation of GSK3β is involved in hirsutine-mediated mPTP opening, ATP depletion, and apoptosis

**Fig. 4:**
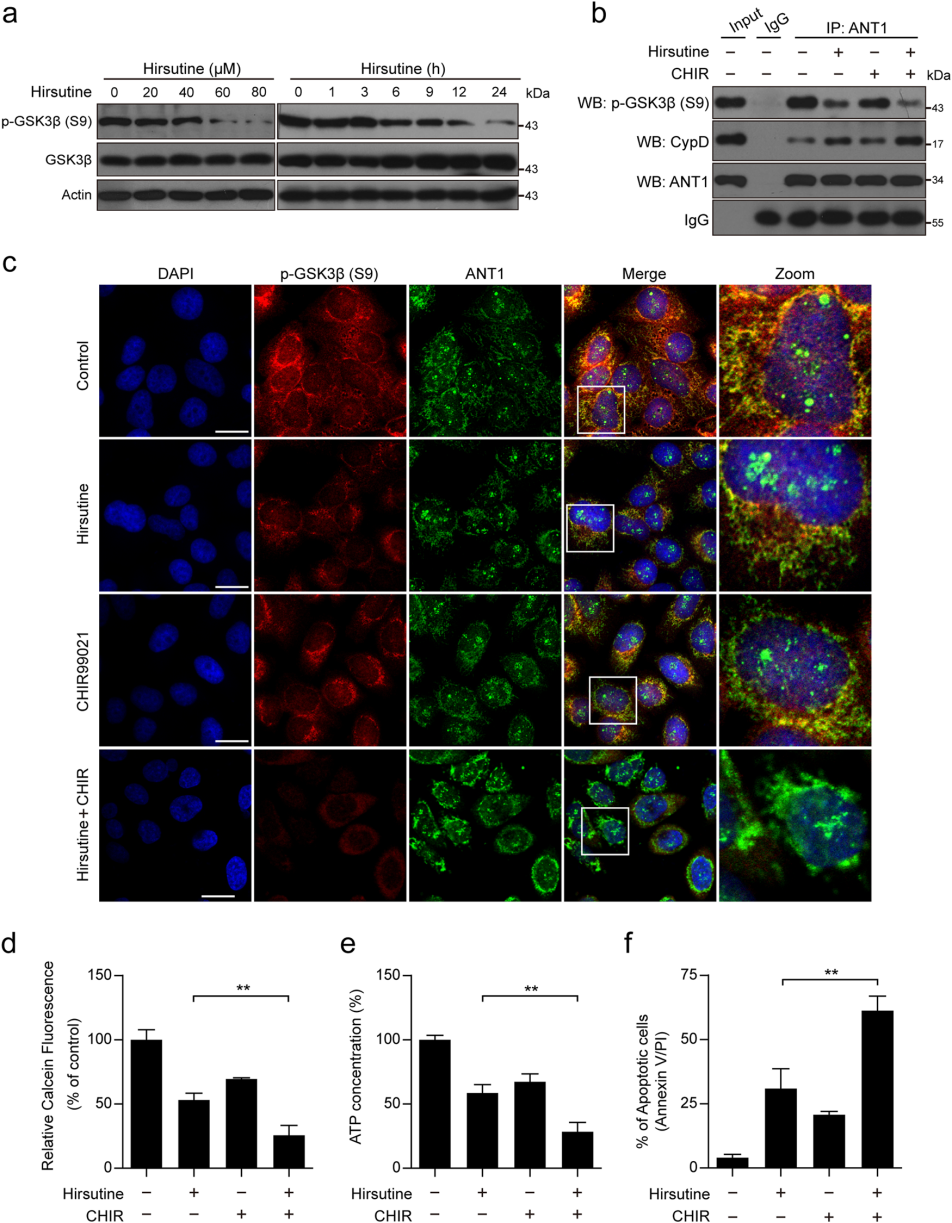
GSK3β inhibitor CHIR potentiates hirsutine-induced mPTP opening and apoptosis. **a** A549 cells were treated with various concentrations of hirsutine for 24 h or 80 μM hirsutine for different time intervals; the expressions of GSK3β and p-GSK3β were determined by immunoblotting. **b** A549 cells were pretreated with 5 µM CHIR99021 (CHIR, a GSK3 inhibitor) for 2 h, followed by treatment with 60 μM hirsutine for 24 h. Whole-cell lysates were prepared and subjected to immunoprecipitation to determine the interaction of p-GSK3β, CypD, and ANT1. **c** After treatment as indicated in **b**, the cells were stained with p-GSK3β (Alexa Fluor 647, red), ANT1 (Alexa Fluor 488, green), and DAPI; images were captured by confocal microscope. Scale bar represents 20 μm. **d** Cells were treated as indicated in **b**, the calcein fluorescence in the mitochondria was analyzed by microplate reader. **e** Cells were treated as indicated in **b**, ATP concentrations were measured by using ATP Determination Kit. **f** Cells were treated as indicated in **b**, the percentage of apoptotic cells was determined by flow cytometry using Annexin V-FITC/PI staining. Data are expressed as the mean ± SD (*n* = 3), **P < 0.01

It has been shown that GSK3β activity is a determinant of the threshold for mPTP opening, the interaction of ANT with CypD triggers mPTP opening, and binding of phospho-GSK3β to ANT1 suppresses the interaction of ANT1 with CypD^18,39^. Our western blot analyses showed that treating A549 cells with hirsutine resulted in decreases in levels of phospho-GSK3β (Ser9) in a dose- and time-dependent manner (Fig. 4a). The immunoprecipitation assay showed that hirsutine treatment decreased the interaction of phospho-GSK3β and ANT1, and pretreating cells with GSK3 inhibitor CHIR obviously enhanced hirsutine-mediated inhibition of phospho-GSK3β/ANT1 interaction. In contrast, treating cells with hirsutine resulted in an increase in the interaction of ANT1 with CypD, and pretreating cells with CHIR enhanced this ANT1/CypD interaction mediated by hirsutine (Fig. 4b). An immunofluorescence assay also confirmed that a reduction in the colocalization of phospho-GSK3β and ANT1 in A549 cells treated with hirsutine was observed compared to that in the control cells. Pretreatment with CHIR further reduced the colocalization of phospho-GSK3β and ANT1 compared to that in hirsutine-treated cells (Fig. 4c). Furthermore, pretreatment with CHIR further promoted the opening of mPTP, depletion of ATP, as well as induction of apoptosis mediated by hirsutine (Fig. 4d–f). Taken together, these findings indicate that dephosphorylation of GSK3β is involved in hirsutine-mediated mPTP opening through ANT1/CypD interaction, leading to depletion of ATP and mitochondrial apoptosis.

### Inhibition of PI3K activity plays an important functional role in hirsutine-induced GSK3β dephosphorylation, mPTP opening, and apoptosis

**Fig. 5:**
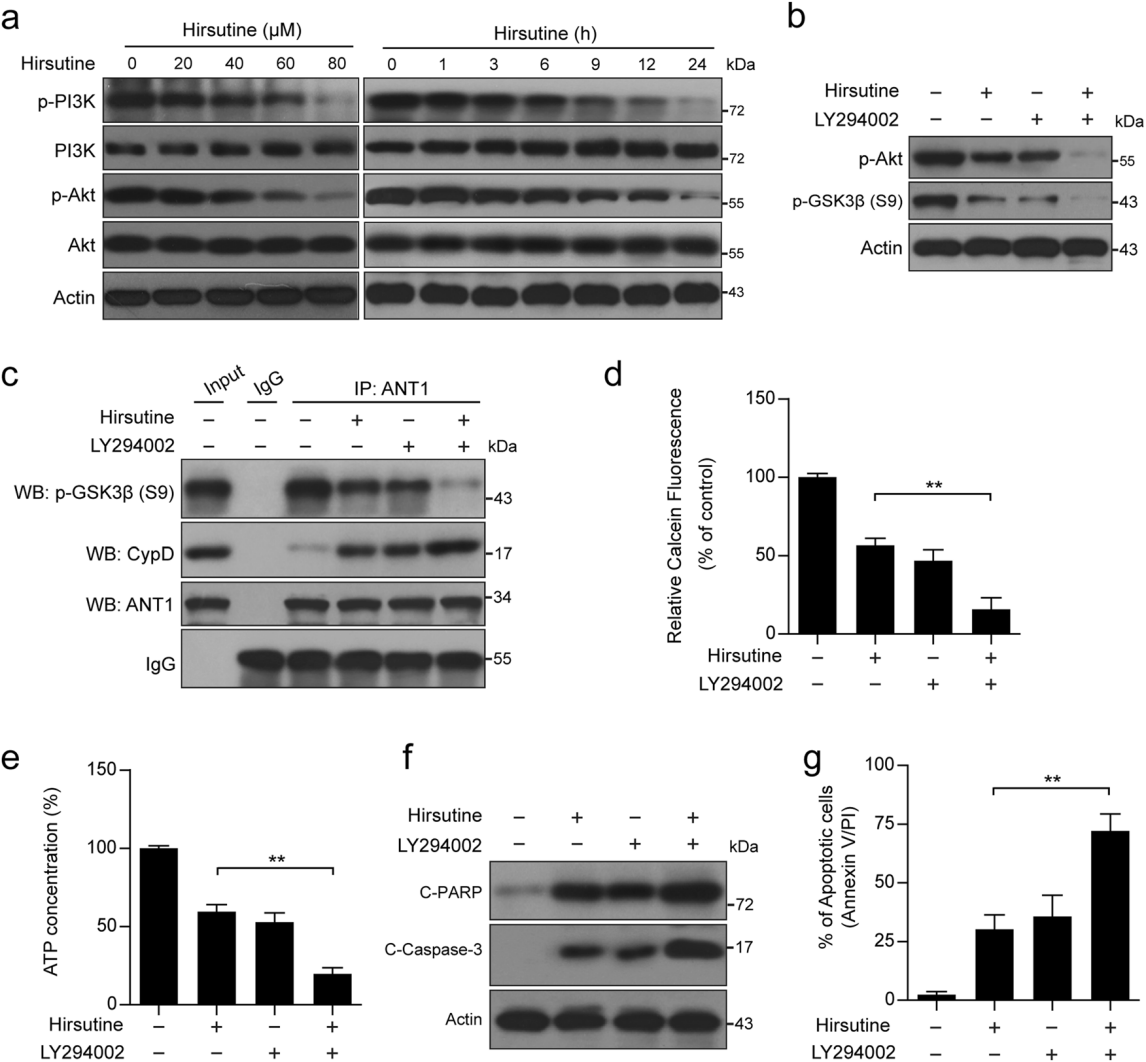
Inhibition of PI3K activity plays an important functional role in hirsutine-induced GSK3β dephosphorylation, mPTP opening, and apoptosis. **a** A549 cells were treated with various concentrations of hirsutine for 24 h or 80 μM hirsutine for different time intervals; whole-cell lysates were prepared and subjected to western blot analysis. **b** A549 cells were pretreated with 20 µM LY294002 (a specific PI3K inhibitor) for 2 h, followed by treatment with 60 μM hirsutine for 24 h. Whole-cell lysates were prepared and subjected to western blot analysis. **c** Cells were treated as indicated in **b**, the interaction of p-GSK3β, CypD, and ANT1 was determined by immunoprecipitation. **d** Cells were treated as indicated in **b**, the calcein fluorescence in the mitochondria was analyzed by microplate reader. **e** Cells were treated as indicated in **b**, ATP concentrations were measured by using ATP Determination Kit. **f** Cells were treated as indicated in **b**, C-PARP and C-Caspase 3 in whole-cell lysates were determined by immunoblotting. **g** Cells were treated as indicated in **b**, the percentage of apoptotic cells was determined by flow cytometry using Annexin V-FITC/PI staining. Data are expressed as the mean ± SD (*n* = 3), **P < 0.01

Since GSK3β is a major downstream target of PI3K/Akt signaling^40–42^, we therefore determined the effects of hirsutine on phosphorylation of PI3K and Akt. Treatment of A549 cells with hirsutine resulted in decreases in levels of phospho-PI3K (p-PI3K) and phospho-Akt (p-Akt) in a dose- and time-dependent manner (Fig. 5a). To further confirm the functional role of PI3K/Akt in hirsutine-induced apoptosis, a specific PI3K inhibitor LY294002 was employed. Pretreatment with LY294002 markedly enhanced hirsutine-mediated Akt inactivation and GSK3β dephosphorylation (Fig. 5b). Furthermore, pretreatment with LY294002 significantly enhanced hirsutine-mediated inhibition of phospho-GSK3β/ANT1 interaction, resulting in an increase in the association between CypD and ANT1, causing mPTP opening, PARP degradation, and caspase 3 activation, culminating in cell apoptosis (Fig. 5c–g). These findings indicate that inactivation of PI3K/Akt plays a critical role in hirsutine-mediated GSK3β dephosphorylation, mPTP opening, and mitochondrial apoptosis.

### ROCK1/PTEN/PI3K signaling pathway plays a crucial role in hirsutine-mediated GSK3β dephosphorylation and mitochondrial apoptosis

**Fig. 6:**
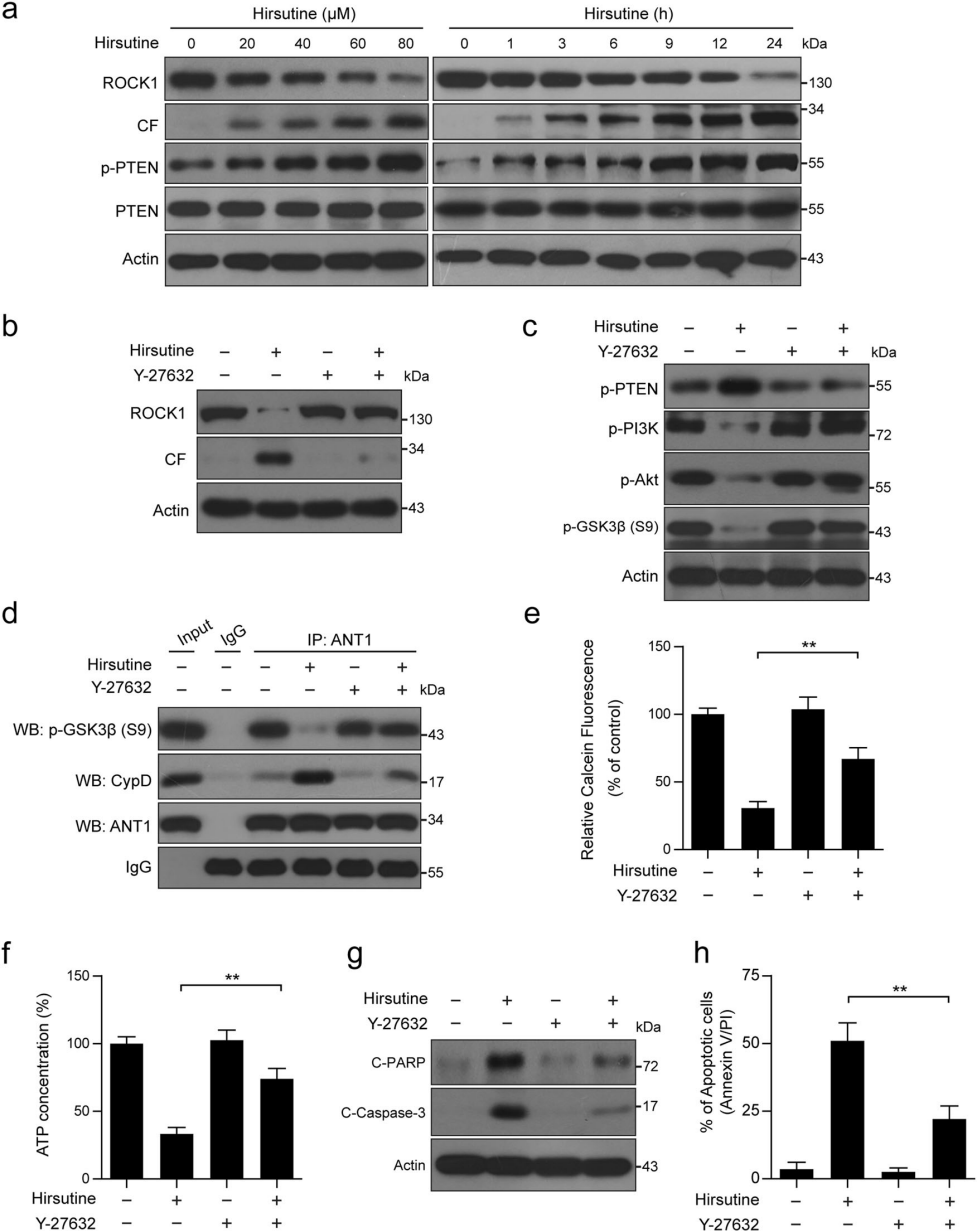
ROCK1/PTEN/PI3K pathway regulates hirsutine-mediated GSK3β dephosphorylation, mPTP opening, and apoptosis. **a** A549 cells were treated with various concentrations of hirsutine for 24 h or 80 μM hirsutine for different time intervals; whole-cell lysates were prepared and subjected to western blot analysis. CF: cleavage fragment. **b**, **c** A549 cells were pretreated with 20 µM Y-27632 (a ROCK1 activation inhibitor) for 2 h, followed by treatment with 80 μM hirsutine for 24 h. Whole-cell lysates were prepared and subjected to western blot analysis. **d** Cells were treated as indicated in **b**, the interaction of p-GSK3β, CypD, and ANT1 was determined by immunoprecipitation. **e** Cells were treated as indicated in **b**, the calcein fluorescence in the mitochondria was analyzed by microplate reader. **f** Cells were treated as indicated in **b**, ATP concentrations were measured by using ATP Determination Kit. **g** Cells were treated as indicated in **b**, C-PARP and C-Caspase 3 in whole-cell lysates were determined by immunoblotting. **h** Cells were treated as indicated in **b**, the percentage of apoptotic cells was determined by flow cytometry using Annexin V-FITC/PI staining. Data are expressed as the mean ± SD (*n* = 3), **P < 0.01

It has recently been reported that PTEN is a negative regulator of PI3K/Akt pathway, and ROCK1 cleavage/activation plays a critical role in the regulation of PTEN activation^43,44^. We then examined the effects of hirsutine on PTEN and ROCK1 activation. Treatment of A549 cells with hirsutine resulted in decreased ROCK1 levels and increased ROCK1 cleavage and increased phosphorylated PTEN (p-PTEN) levels in a dose- and time-dependent manner (Fig. 6a).

We also investigated the effects of caspase inhibition on hirsutine-induced ROCK1 activation, PTEN phosphorylation, and PI3K/Akt/GSK3β dephosphorylation. Western blot analysis showed that pretreatment with the broad spectrum caspase inhibitor z-VAD-fmk blocked hirsutine-induced caspase-3 activation and PARP degradation, but failed to prevent ROCK1 activation, PTEN phosphorylation, and PI3K/Akt/GSK3β dephosphorylation (Figure S2). Such findings suggest that hirsutine-mediated perturbations in ROCK1/PTEN/PI3K/Akt signaling proceeded through a caspase-independent pathway.

To further confirm the function role of ROCK1 activation in the regulation of PTEN/PI3K/Akt signaling, GSK3β dephosphorylation, and mitochondrial apoptosis, a ROCK1 inhibitor Y27632 was employed. Western blot analysis indicated that pretreatment of A549 cells with Y27632 abrogated hirsutine-induced ROCK1 cleavage/activation (Fig. 6b). Pretreatment of A549 cells with Y27632 also attenuated hirsutine-mediated activation of PTEN, inactivation of PI3K/Akt, and dephosphorylation of GSK3β (Fig. 6c). Furthermore, pretreatment of A549 cells with Y27632 attenuated hirsutine-mediated inhibition of phospho-GSK3β/ANT1 interaction, ANT1/CypD interaction, mPTP opening, ATP depletion, PARP degradation, and caspase 3 activation, as well as apoptosis (Fig. 6d–h).

To further confirm these results, a lentivirus shRNA approach was used to stably knockdown ROCK1 expression (Supplementary Figure S3a). Knockdown of ROCK1 significantly blocked hirsutine-mediated activation of PTEN, inactivation of PI3K/Akt, and dephosphorylation of GSK3β (Supplementary Figure S3b). Knockdown of ROCK1 also blocked hirsutine-mediated inhibition of phospho-GSK3β/ANT1 interaction, ANT1/CypD interaction, mPTP opening, ATP depletion, PARP degradation, and caspase 3 activation, as well as apoptosis (Supplementary Figures S3c-g). Together, these findings suggest that ROCK1/PTEN/PI3K/Akt signaling pathway plays a crucial role in hirsutine-mediated GSK3β dephosphorylation, ATP depletion, and mitochondrial apoptosis.

### Hirsutine inhibits tumor growth and induces apoptosis in an A549 xenograft mouse model through ROCK1/PTEN/PI3K/Akt/GSK3β signaling pathway

**Fig. 7:**
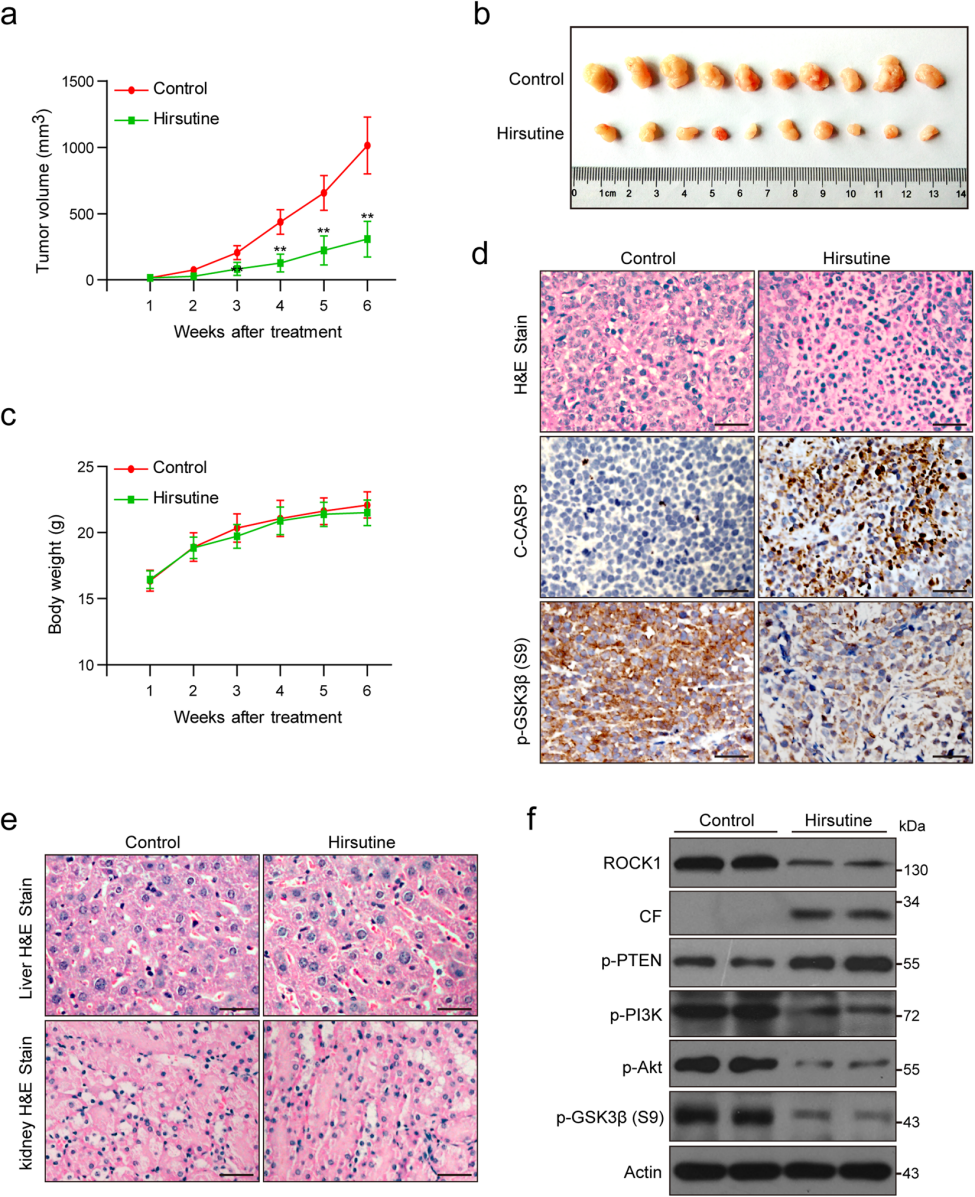
Hirsutine inhibits tumor growth and induces apoptosis in A549 xenograft model. Mice were subcutaneously inoculated with A549 cells (2 × 10^6^) into the right flanks and randomly divided into two groups (*n* = 10). Mice were injected intraperitoneally (i.p.) with 10 mg/kg/day hirsutine or an equal volume of vehicle. **a** The tumor growth curve of vehicle control mice and hirsutine-treated mice. **P < 0.01 vs. the control group. **b** Representative image of tumors of two groups. **c** There were no significant differences of body weights between vehicle control and hirsutine-treated groups. **d** Representative two tumor tissues from each group were fixed and subjected to hematoxylin and eosin (H&E) staining, and immunohistochemistry staining for C-CASP3 and p-GSK3β (Ser9). **e** Representative H&E-stained liver and kidney sections from two groups are shown. Scale bars: 25 μm. **f** Representative two tumor tissues from each group were prepared and subjected to western blot analysis

To determine whether these in vitro findings were applicable in vivo, nude mice were inoculated subcutaneously with A549 cells and injected with either vehicle or hirsutine (10 mg/kg, intraperitoneally). Treatment of mice with hirsutine resulted in a significant suppression of tumor growth after 3 weeks of drug exposure (**P < 0.01 vs. vehicle control) (Fig. 7a, b). Notably, mouse weights did not exhibit major changes in mice treated with hirsutine compared to that in vehicle control (Fig. 7c). To evaluate the effect of hirsutine on morphological changes and induction of apoptosis in excised tumor tissue from A549 xenografts, hematoxylin and eosin (H&E) staining and immunohistochemistry analyses were performed. The sections of A549 xenografts from mice treated with hirsutine exhibited a reduction in densely packed cells and consisted of sparse areas of apoptotic/necrotic cells (Fig. 7d, top panels). Treatment with hirsutine caused an increase in immunoreactivity for cleaved caspase-3, which is indicative of apoptosis (Fig. 7d, middle panels). A decrease in immunoreactivity of phospho-GSK3β was also observed in excised tumor tissue from hirsutine-treated animals (Fig. 7d, bottom panels). In contrast, histopathological examination of liver and kidney revealed no morphological difference between the control and hirsutine-treated animals (Fig. 7e). These findings suggest that hirsutine selectively inhibits tumor growth in A549 xenograft mouse model without causing additional toxic effect on normal tissues like liver and kidney in vivo.

Western blot analysis conducted on excised tumor tissue from treated animals revealed that treatment with hirsutine resulted in cleavage/activation of ROCK1, activation of PTEN, inactivation of PI3K, Akt and dephosphorylation of GSK3β (Fig. 7f). Together, these results indicate that hirsutine effectively inhibits tumor growth in an A549 xenograft mouse model through ROCK1/PTEN/PI3K/Akt signaling-mediated GSK3β dephosphorylation and apoptosis.

To further confirm the function role of PI3K in hirsutine-inhibited tumor growth in vivo, the combined treatment of PI3K inhibitor LY294002 and hirsutine was employed in a mouse xenograft model. As shown in Figure S4a and b, treatment of mice with hirsutine resulted in a significant suppression of tumor growth (**P < 0.01 vs. vehicle control), and co-treatment of mice with LY294002 significantly enhanced hirsutine-mediated suppression of tumor growth (**P < 0.01, combination vs. hirsutine treatment alone). However, mouse weights did not exhibit major changes in mice treated with hirsutine or combination compared to that in vehicle control (Figure S4c). Western blot analysis showed that co-treatment with LY294002 markedly enhanced hirsutine-mediated Akt inactivation, GSK3β dephosphorylation, and caspase 3 activation (Figure S4d).

## Discussion

In the present study, we demonstrated that a natural product hirsutine potently induces mitochondrial injury and caspase-dependent apoptosis in human lung cancer cells. This phenomenon was primarily due to mPTP opening. Dephosphorylation of GSK3β is involved in hirsutine-mediated mPTP opening, ATP depletion, and apoptosis. GSK3 is currently well known as a multi-functional kinase that performs a regulatory role in several cellular functions, including embryonic development, cell metabolism, proliferation, and apoptosis^45^. In NSCLC, GSK3β has been recently reported to be over-expressed in tumor tissue compared to normal tissue and this was found to correlate with poor patient prognosis^46^. Inhibition of GSK3β suppressed tumorigenesis by inhibiting cell proliferation and inducing apoptosis, suggesting that GSK3β could be developed as a tumor promoter and a potential therapeutic target in NSCLC^47^. It has been shown that GSK3β activity is a determinant of the threshold for mPTP opening; one of the mechanisms has suggested that binding phospho-GSK3β to ANT1 suppresses interaction of ANT1 with CypD, inhibiting mPTP opening^18,39^. Consistent with this report, our study demonstrated that treatment of A549 cells with hirsutine caused a dose- and time-dependent decrease in the levels of phospho-GSK3β, dephosphorylation of GSK3β appears to be necessary for hirsutine-mediated mPTP opening, and mitochondrial apoptosis through ANT1/CypD interaction based on the following findings. First, treating cells with hirsutine decreased the interaction and colocalization of GSK3β and ANT1 interaction and increased the interaction of ANT1 and CypD. Second, pretreating cells with GSK3β inhibitor CHIR further promoted hirsutine-inhibited interaction and colocalization of GSK3β and ANT1, and enhanced hirsutine-mediated interaction of ANT1 and CypD. Third, pretreating cells with CHIR significantly enhanced hirsutine-mediated mPTP opening, ATP depletion, and apoptosis. Together, our results demonstrate that hirsutine-mediated inhibition of GSK3β activity is required for mPTP opening, ATP depletion, as well as mitochondrial apoptosis.

Our results provide detailed information on the molecular mechanisms by which hirsutine induces mitochondrial apoptosis in human lung cancer cells (i.e., by activation/cleavage of ROCK1, activation of PTEN, and inactivation of PI3K/Akt). ROCK belongs to a family of serine/threonine kinases and plays a central role in the regulation of cell adhesion, migration, proliferation, and apoptosis^19^. Elevated ROCK expression has been detected in several human cancers, which correlated with poor outcome^48^. ROCK1 can be activated by cleavage via caspases and RhoA during apoptosis, generating a constitutive active fragment, leading to the downstream substrate PTEN phosphorylation and activation^21,49^. Cotreatment with the pan-caspase inhibitor z-VAD-fmk, which abrogated hirsutine-induced activation of caspase-3 and apoptosis, failed to prevent ROCK1/PTEN activation. These data strongly suggest that factors other than caspase-dependent events are involved in this phenomenon. PTEN is a tumor suppressor gene that is often mutated in several human cancers^21^. PTEN inactivates PI3K/Akt by dephosphorylating PIP3 to PIP2, resulting in Akt downstream targets GSK3β dephosphorylation at Ser 9^50^. It is more likely that activation of PTEN and inactivation of the PI3K/Akt cascade acts downstream of the ROCK1 pathway during hirsutine-induced mPTP opening and mitochondrial apoptosis based on the following evidence. First, treatment of A549 cells with hirsutine resulted in activation/cleavage of ROCK1, activation of PTEN, and inactivation of PI3K/Akt in a dose- and time-dependent manner. Second, pretreatment with PI3K inhibitor LY294002 enhanced hirsutine-mediated Akt/GSK3β dephosphorylation, inhibition of phospho-GSK3β/ANT1 interaction, ANT1/CypD interaction, mPTP opening, ATP depletion, PARP degradation, and caspase 3 activation, as well as apoptosis. Third, knockdown of ROCK1 or pretreatment of cells with the ROCK1 inhibitor Y27632 dramatically abrogated hirsutine-mediated PTEN activation, PI3K/Akt inactivation, GSK3β dephosphorylation, ANT1/CypD interaction, mPTP opening, ATP depletion, PARP degradation, and caspase 3 activation, as well as apoptosis. Taken together, our findings indicate that ROCK1/PTEN/PI3K/Akt signaling pathway plays a crucial role in the regulation of GSK3β dephosphorylation, mPTP opening, and mitochondrial apoptosis mediated by hirsutine.

Our in vivo studies have shown that hirsutine markedly inhibited tumor growth in A549 xenograft mouse model, and co-treatment with LY294002 significantly enhanced hirsutine-mediated suppression of tumor growth. The mechanistic studies in vivo indicated that ROCK1/PTEN/PI3K/Akt signaling pathway-regulated GSK3β dephosphorylation and apoptosis are involved in hirsutine-inhibited tumor growth in an A549 xenograft mouse model. These results suggest that sufficient hirsutine regimen can be achieved in vivo to recapitulate in vitro actions. Of note, little toxicity was observed in animals with the doses and schedules used in these studies.

**Fig. 8:**
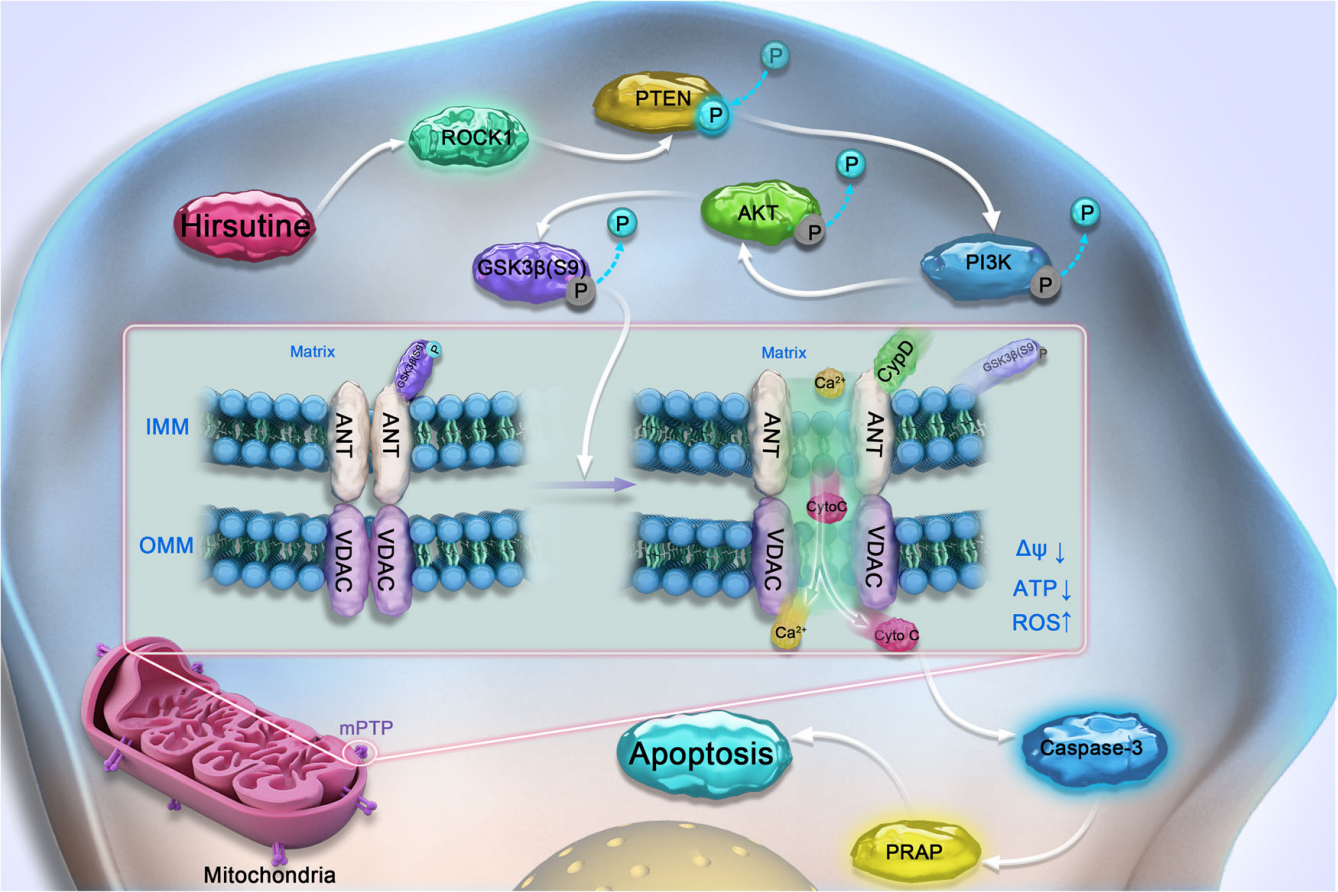
Model for hirsutine-induced apoptosis in human lung cancer cells. Hirsutine induces ROCK1 cleavage/activation, PTEN activation (phosphorylation), and PI3K/Akt inactivation (dephosphorylation), resulting in GSK3β dephosphorylation, leading to decreases in the binding of GSK3β and ANT1 and increases in the interaction of ANT1 to CypD, culminating in mPTP opening, caspase activation, and apoptosis

In summary, the present studies indicate that hirsutine effectively induces mitochondrial apoptosis in A549 cells and inhibits tumor growth in an A549 xenograft mouse model. Collectively, these findings suggest a hierarchy model of hirsutine-induced apoptosis in human lung cancer cells in which hirsutine-induced ROCK1 activation represents a primary event resulting in PTEN activation and PI3K/Akt inactivation, in turn, leading to GSK3β dephosphorylation and mPTP opening, and culminating in caspase-3 activation and apoptosis (Fig. 8). These findings could provide a novel mechanistic basis for the application of hirsutine in the treatment of human lung cancer.

## Materials and methods

### Reagents

Hirsutine (A1067) was purchased from Chengdu Must Biotechnology Company (Chengdu, China); CSA (HY-B0579) was purchased from Medchem Express; CHIR-99021 (S2924), z-VAD-fmk (S7023), LY294002 (S1105), and Y-27632 (S1049) were purchased from Selleckchem.

### Cell culture

The human lung carcinoma cell line A549 and NCI-H1299 and human lung fibroblast cell line WI38 were purchased from the American Type Culture Collection (ATCC), human normal hepatocytes cell line LO2 was purchased from the cell bank of the Chinese Academy of Sciences. A549, NCI-H1299, and LO2 cells were cultured in Dulbecco’s modified Eagle’s medium (DMEM, Invitrogen, 12491) supplemented with 10% fetal bovine serum (FBS, Gibco, 10100). WI38 cells was cultured in Minimum Essential Medium (MEM, Invitrogen, 11090081) supplemented with 10% FBS, 0.1 mM non-essential amino acids (Invitrogen, 11140), 1 mM sodium pyruvate (Invitrogen, 11360070), 2 mM GlutaMAX (Invitrogen, 35050).

### Plasmids and establishment of stable cell lines

Human ROCK1 shRNA (target sequence: 5′-CCGGGCACCAGTTGTACCC GATTTACTCGAGTAAATCGGGTACAACTGGTGCTTTTTG-3′), non-specific control shRNA plasmid (sc-108060), human Bax shRNA (sc-29212), and Bak shRNA (sc-29786) were purchased from Santa Cruz Biotechnology and used for gene silencing. Stable cell lines were established by using a lentiviral system as our previously described^51^.

### Cell viability assay

Cell viability assay was performed with a Cell Counting Kit-8 (CCK-8, Dojindo Laboratories, CK04) according to the manufacturer’s instructions. Briefly, cells were seeded in 96-well plates and treated with hirsutine, the CCK-8 solutions were added and incubated for 2 h. Absorption at 450 nm was subsequently measured on a microplate reader (Thermo, Varioskan Flash). Each experiment was repeated three times individually. The results were expressed as a percentage of control, which was set at 100 %.

### ATP luminescence assay

The ATP levels were measured using a firefly luciferase-based ATP Determination Kit (Beyotime, S0026). Cells were lysed and centrifuged at 12,000×*g* for 5 min, the supernatant was mixed with 100 μL ATP detection working solution, and the luminescence value was measured by using a microplate reader (Thermo, Varioskan Flash). The ATP level was expressed as the percentage of the level that was observed in the control groups.

### Measurement of mitochondrial membrane potential

The mitochondrial membrane potential was measured by using a JC-1 kit (Beyotime Company, C2006) according to the manufacturer’s instructions. Briefly, cells were seeded in 96-well plates or 24-well plates; after treatment with hirsutine, the cells were incubated with 1× JC-1 reagent solution for 15 min. Next, washed twice by 1× assay buffer, the fluorescence was analyzed by using LSM780 confocal laser scanning microscope (Zeiss, Germany) or microplate reader (Thermo, Varioskan Flash) at 590 nm (red) and 530 nm (green). The fluorescence ratio of JC-1 aggregates (red) to JC-1 monomers (green) is an index of the mitochondrial membrane potential.

### ROS detection

Intracellular ROS level was detected by staining cells with 2,7-dichlorofluorescein diacetate (DCF-DA, Molecular Probes, C6827). Briefly, cells were trypsinized and collected by centrifugation, then washed twice by PBS and stained with 10 μM DCF-DA in Hank’s balanced salt solution (HBSS) for 20 min. The stained cells were washed with PBS and analyzed by flow cytometry (FACScan, Becton Dickinson) with an excitation wavelength of 488 nm and an emission wavelength of 525 nm.

### Apoptosis determination

Cells were trypsinized and collected, and then washed twice with cold PBS. Cells were resuspended in 100 μL 1× binding buffer with 5 µL Annexin V-FITC and 5 µL propidium iodide (PI) (BD Biosciences, 556547). After incubation for 15 min at 25 °C in the dark, additional 200 µL 1× binding buffer was added in each tube and analyzed by flow cytometry (FACScan, Becton Dickinson).

### Assessment of mPTP opening

mPTP opening was assessed by using the calcein-AM/cobalt method as previously reported. Cells were seeded in 96-well plates or 24-well plates; after treatment with hirsutine, the cells were washed with PBS and loaded with 5 µM Calcein-AM and 0.5 mM CoCl_2_ (cytosolic calcein quencher) in HBSS for 15 min at 37 °C. After washing twice with PBS, the cells were analyzed by LSM780 confocal laser scanning microscope (Zeiss, Germany) or microplate reader (Thermo, Varioskan Flash) with an excitation wavelength of 488 nm and an emission wavelength of 525 nm.

### Transmission electron microscopy

Harvested cells were fixed with 2.5% glutaraldehyde in PBS at 4 °C overnight, post-fixed in 2% osmium tetroxide in cacodylate buffer for 2 h, dehydrated through a graded series (50, 70, 90, and 100%) of ethanol and embedded in Epon. Then samples were sectioned, stained with uranyl acetate and lead citrate, and finally observed under a Hitachi-7500 transmission electron microscopy.

### Immunoprecipitation and western blot analysis

Cells were harvested and lysed, ANT1 antibody was added to the lysates and rotated overnight at 4 °C, and then incubated with protein A/G agarose beads (Pierce, 88802) for 3 h, immunoprecipitates were washed five times with PBS. Protein lysates and immunoprecipitated proteins were separated by SDS-PAGE and transferred to PVDF membranes; the membranes were blocked and incubated with appropriate primary antibodies at 4 °C overnight. The following antibodies were used: cleaved PARP (5625, 1:500), cleaved caspase-3 (9661, 1:500), p-GSK3β (Ser9) (5558, 1:1000), GSK3β (12456, 1:2000), p-PTEN (9554, 1:1000), PTEN (9559, 1:1000), PI3K (4257, 1:1000), p-PI3K (4228, 1:1000), Akt (9272, 1:2000), p-Akt (9271, 1:1000), Bak (3814, 1:1000), and Bim (2933, 1:1000) were purchased from Cell Signaling Technology; Cyto C (sc-13156, 1:5000), CypD (sc-376061, 1:1000), Bax (sc-7480, 1:500), Bcl-2 (sc-509, 1:500), Bcl-xl (sc-8392, 1:500), Mcl-1 (sc-12756, 1: 500), Noxa (sc-56169, 1:250), and Puma (sc-374223, 1:250) were from Santa Cruz Biotechnology; ANT1 (ab102032, 1:1000), ROCK1 (ab45171, 1:1000) was from Abcam. Rabbit IgG (A7016, 1:1000) was from Beyotime; Actin (A1978, 1:50,000) was purchased from Sigma. The membranes were then incubated with horseradish peroxidase (HRP)-conjugated goat anti-rabbit (KPL, 074-1516) or goat anti-mouse (KPL, 074-1802) secondary antibody for 2 h. The protein signals were visualized by enhanced chemiluminescence (Bio-Rad, 170-5061).

### Fluorescence microscopy

Cells were grown on glass coverslips overnight. After treatment with hirsutine, the cells were fixed with 4% paraformaldehyde for 10 min, permeabilized with 0.1% Triton X-100 for 5 min, blocked with 5% BSA for 30 min, and then incubated with ANT1 and p-GSK3β at 4 °C overnight followed by incubation with Alexa Fluor 488 donkey anti-rabbit IgG (A11001, 1:300) and Alexa Fluor 647 donkey anti-mouse IgG (A31573, 1:300) (Molecular Probes) at 37 °C for 1 h. Cells were counterstained with DAPI (C1005, Beyotime), and images were taken with a LSM780 confocal laser scanning microscope (Zeiss, Germany).

### Tumor xenografts

All animal experiments were approved by the Animal Care and Use Committee of Third Military Medical University. Nude mice were purchased from Vital River Laboratories (VRL, Beijing, China). Mice were subcutaneously inoculated with A549 cells (resuspended in DMEM/Matrigel 1:1 (v/v)) into the right flanks. One week after tumor inoculation, the mice were injected intraperitoneally (i.p.) with 5 or 10 mg/kg/day hirsutine (dissolved in propylene glycol/0.9% physiological saline 3:7) and/or LY294002 (25 mg/kg/daily) or an equal volume of vehicle. Tumor volume and body weight were measured and tumor volume was calculated by the formula: volume = (length × width^2^)/2. The mice were euthanized by cervical dislocation at the termination of the experiment (42 days after treatment in Fig. 7, 30 days after treatment in Figure S4). Tumor tissues were harvested, representative tumor tissues from each group were lysed rapidly and subjected to western blot analysis. Tumor, liver, and kidney tissues were fixed in paraformaldehyde for 7 days, paraffin-embedded tissues were sectioned and analyzed by H&E staining and immunohistochemical analysis.

### Statistical analysis

Data are expressed as the mean ± SD from at least three separate experiments. The comparisons were performed using one-way analysis of variance (ANOVA) in conjunction with Tuckey and Dunnett tests. *P < 0.05 and **P < 0.01 were considered statistically significant.

## Electronic supplementary material

Supplementary Information
