# Supplementary material for: Hirsutine induces mPTP-dependent apoptosis through ROCK1/PTEN/PI3K/GSK3β pathway in human lung cancer cells
Source: Cell Death Dis. 2018 May 22;9(6):598. doi: 10.1038/s41419-018-0641-7 (PMC5964100; doi:10.1038/s41419-018-0641-7)
Supplement: Supplementary file 1 — Supplementary Information [file 41419_2018_641_MOESM1_ESM.docx]

**Supplementary Information**

**Hirsutine induces mPTP-dependent apoptosis through** **ROCK1/PTEN/PI3K/GSK3β pathway in human lung cancer cells**

Rong Zhang, Guobing Li, Qian Zhang, Qin Tang, Jingbin Huang, Changpeng Hu, Yali Liu, Qing Wang, Wuyi Liu, Ning Gao and Shiwen Zhou


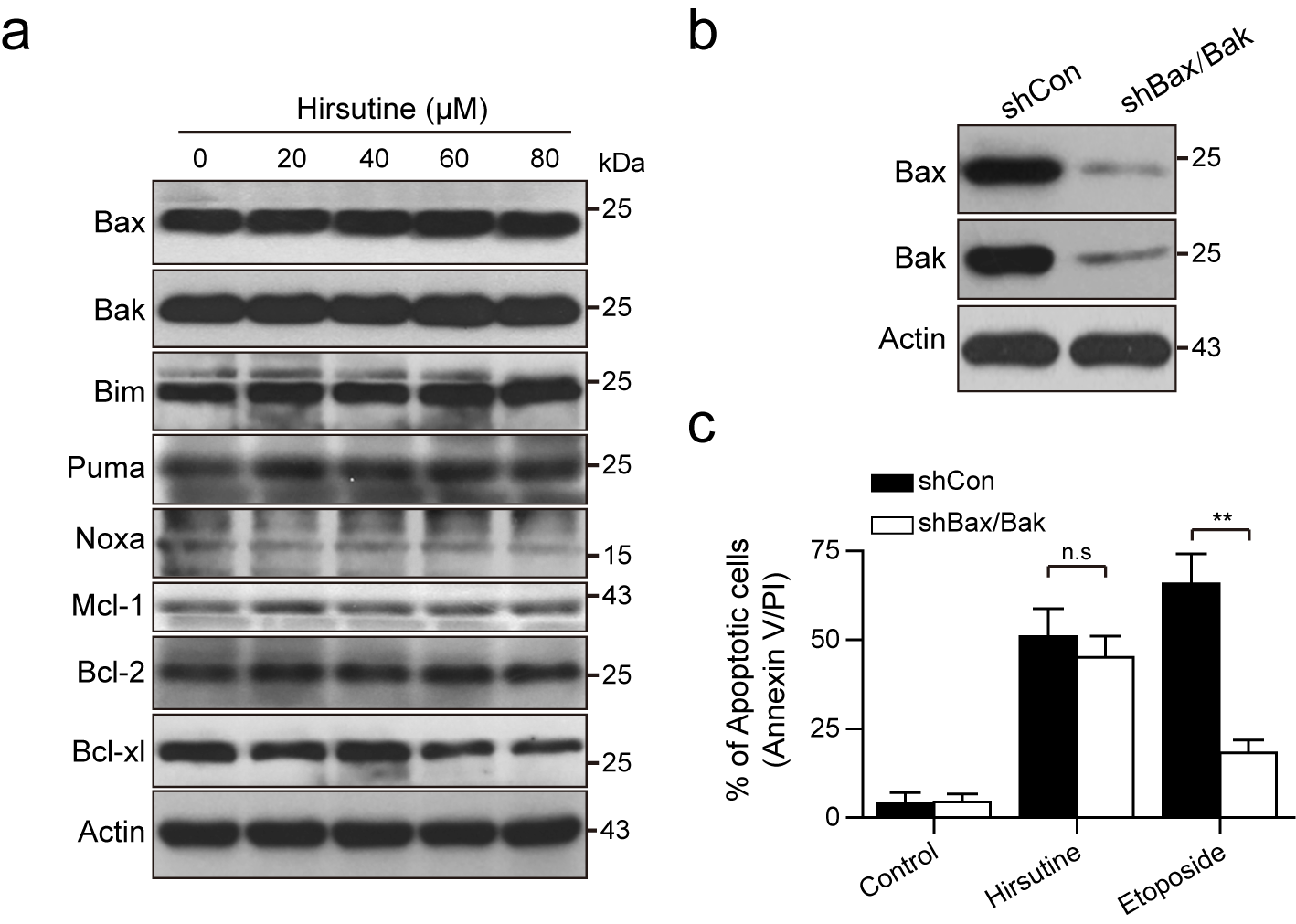


**Figure S1.** Bax and Bak are not the key regulators in hirsutine-induced apoptosis in A549 cells. (**a**) A549 cells were treated with various concentrations of hirsutine for 24 h, whole cell lysates were prepared and subjected to western blot analysis. (**b** and **c**) A549 cells transfected with shCon or co-transfected with shBax and shBak plasmids, followed by treating with hirsutine (80 μM) or etoposide (100 μM) for 24 h. The expression of Bax and Bak was detected by western blot. The percentage of apoptotic cells was determined by flow cytometry using Annexin V-FITC/PI staining. Data are expressed as the mean ± SD (n=3), n.s, not significant, ***P* < 0.01.


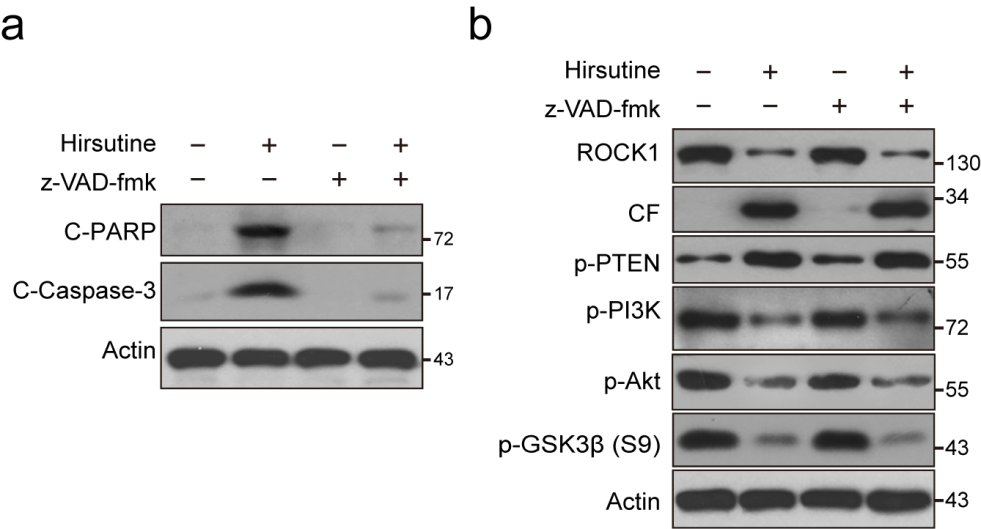


**Figure S2.** Hirsutine-mediated interruption of ROCK1/PTEN/PI3K/Akt signaling proceeded through a caspase-independent pathway. (**a** and **b**) A549 cells were pretreated with the caspase inhibitor z-VAD-fmk (10 μM) for 2 h, followed by treatment with 80 μM hirsutine for 24 h. whole cell lysates were prepared and subjected to western blot analysis.


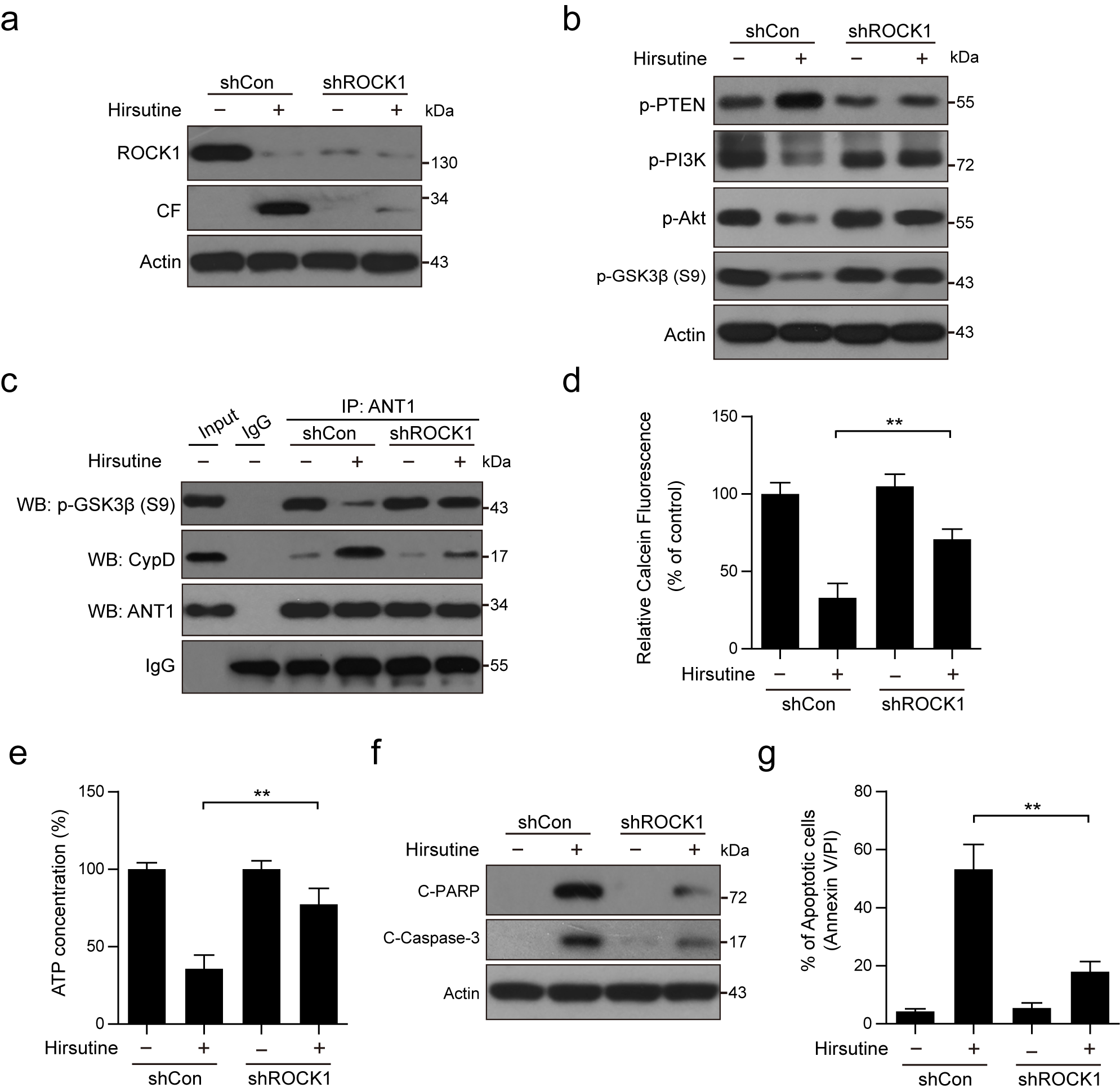


**Figure S3.** Knockdown of ROCK1 blocked hirsutine-mediated PTEN activation, PI3K/Akt inactivation, GSK3β dephosphorylation and mPTP-dependent apoptosis. (**a** and **b**) shCon and shROCK1 stable cell lines were treated with 80 μM hirsutine for 24 h. Whole cell lysates were prepared and subjected to western blot analysis. (**c**) The interaction of p-GSK3β, CypD and ANT1 were determined by immunoprecipitation. (**d**) The calcein fluorescence in the mitochondria was analyzed by microplate reader. (**e**) ATP concentrations were measured by using ATP Determination Kit. (**f**) C-PARP and C-Caspase 3 in whole cell lysates were determined by immunoblotting. (**g**) The percentage of apoptotic cells was determined by flow cytometry using Annexin V-FITC/PI staining. Data are expressed as the mean ± SD (n=3), ***P* < 0.01.


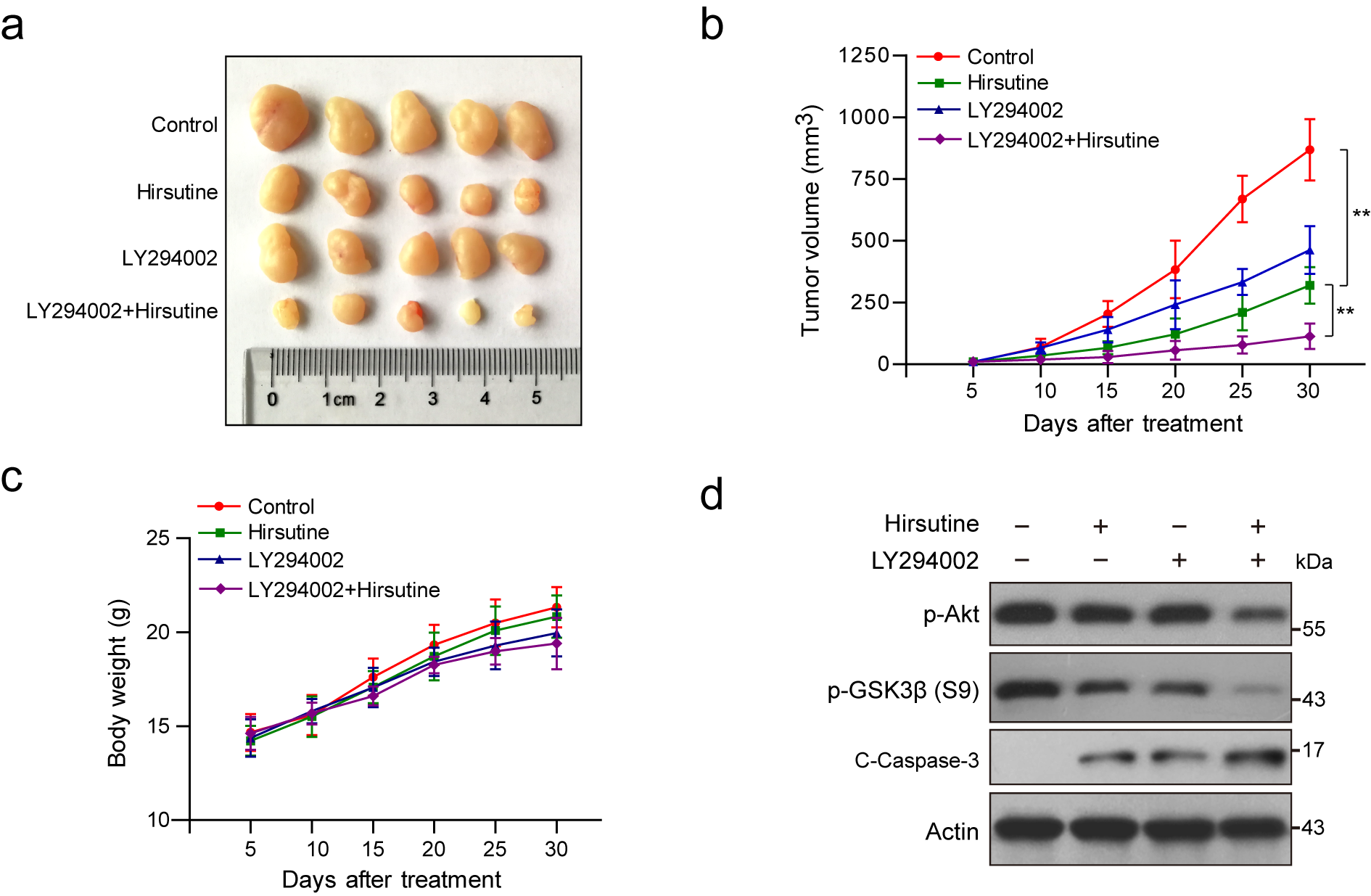


**Figure S4.** LY294002 enhanced hirsutine-mediated suppression of tumor growth *in vivo*. Mice were subcutaneously inoculated with A549 cells (1×10^7^) into the right flanks and randomly divided into four groups (n=5). Hirsutine (5 mg/kg) and/or LY294002 (25 mg/kg) was administered daily by intraperitoneal injection. (**a**) Representative image of tumors in vehicle control mice, mice treated with hirsutine or LY294002 alone or a combination of hirsutine and LY294002. (**b**) Average tumor volume in four groups. **P* < 0.01 vs. the hirsutine treatment alone at the end of the treatment period. (**c**) Body weight changes of mice during 30 days of drug exposure. There were no significant differences of body weights between combination of hirsutine with LY294002 and other groups. (**d**) Representative tumor tissues form each group were prepared and subjected to western blot analysis.
